# User Perceptions of Different Electronic Cigarette Flavors on Social Media: Observational Study

**DOI:** 10.2196/17280

**Published:** 2020-06-24

**Authors:** Xinyi Lu, Long Chen, Jianbo Yuan, Joyce Luo, Jiebo Luo, Zidian Xie, Dongmei Li

**Affiliations:** 1 University of Rochester Rochester, NY United States; 2 Princeton University Princeton, NJ United States; 3 University of Rochester Medical Center Rochester, NY United States

**Keywords:** e-cigarette, flavor, social media

## Abstract

**Background:**

The number of electronic cigarette (e-cigarette) users has been increasing rapidly in recent years, especially among youth and young adults. More e-cigarette products have become available, including e-liquids with various brands and flavors. Various e-liquid flavors have been frequently discussed by e-cigarette users on social media.

**Objective:**

This study aimed to examine the longitudinal prevalence of mentions of electronic cigarette liquid (e-liquid) flavors and user perceptions on social media.

**Methods:**

We applied a data-driven approach to analyze the trends and macro-level user sentiments of different e-cigarette flavors on social media. With data collected from web-based stores, e-liquid flavors were classified into categories in a flavor hierarchy based on their ingredients. The e-cigarette–related posts were collected from social media platforms, including Reddit and Twitter, using e-cigarette–related keywords. The temporal trend of mentions of e-liquid flavor categories was compiled using Reddit data from January 2013 to April 2019. Twitter data were analyzed using a sentiment analysis from May to August 2019 to explore the opinions of e-cigarette users toward each flavor category.

**Results:**

More than 1000 e-liquid flavors were classified into 7 major flavor categories. The fruit and sweets categories were the 2 most frequently discussed e-liquid flavors on Reddit, contributing to approximately 58% and 15%, respectively, of all flavor-related posts. We showed that mentions of the fruit flavor category had a steady overall upward trend compared with other flavor categories that did not show much change over time. Results from the sentiment analysis demonstrated that most e-liquid flavor categories had significant positive sentiments, except for the beverage and tobacco categories.

**Conclusions:**

The most updated information about the popular e-liquid flavors mentioned on social media was investigated, which showed that the prevalence of mentions of e-liquid flavors and user perceptions on social media were different. Fruit was the most frequently discussed flavor category on social media. Our study provides valuable information for future regulation of flavored e-cigarettes.

## Introduction

### Background

An electronic cigarette (e-cigarette) is a product for users to consume nicotine-related aerosols by heating a solution comprising propylene glycol or glycerol, nicotine, and flavoring chemicals [1]. The first e-cigarette product appeared in the market in 2003 and became popular in the United States in 2006-2007 [2]. E-cigarette products are commonly used as substitutes for conventional cigarettes. Consequently, conventional cigarette consumption in the United States has been decreasing in recent years [3]. The number of adult smokers declined from 20.9% in 2005 to 15.5% in 2016 [4]. Recently, people have tended to use e-cigarette products over conventional tobacco products, especially among adolescents and young adults [5]. According to the 2019 National Youth Tobacco Survey, among middle school students, the prevalence of e-cigarette use increased from 4.9% in 2018 to 10.5% in 2019, and for high school students, the number increased from 20.8% in 2018 to 27.5% in 2019 [6]. Approximately 72.2% of high school and 59.2% of middle school exclusive e-cigarette users used flavored e-cigarettes [6]. Flavored e-cigarette use among current young adult users (aged between 18 and 24 years) exceeds that of current older adult users (aged 25 years), and 81.5% of students aged between 12 and 17 years, used e-cigarettes because they came with attractive flavors [7]. Fruit, menthol or mint, candy, desserts, or other sweets were the most popular flavors among high school and middle school students [8].

Although perceived as safe to be ingested, flavorings in e-cigarettes remain to be a health concern, especially for adolescents, as they might not be safe to inhale. Different electronic cigarette liquid (e-liquid) flavors contain different flavoring chemicals [9]. Exposure to e-cigarette aerosols with flavorings could cause DNA damage, increased oxidative stress, inflammatory cytokine release, and epithelial barrier dysfunction [10,11]. A recent study showed that treating monocytic cells with different flavoring chemicals and flavored e-liquids without nicotine could cause different cytotoxicities [11]. E-cigarette flavorings could damage endothelial cells that line the interior of human blood vessels and may increase the risk of heart disease [12]. In particular, cinnamon and menthol flavors were more harmful than other flavors [11]. Diacetyl and 2,3-pentanedione, 2 of the most common flavoring ingredients in e-cigarettes, were found to have adverse effects on lung cells, which can impair lung function and cause a *popcorn lung* [9]. Another study suggested that the complexity of e-cigarettes on human health goes beyond respiratory and cardiac systems and may have significant implications on oral health [13].

### Objective

Although most studies focus on the health effects of different e-liquid flavors, it has become very important to understand the relative proportions of mentions of different e-liquid flavors and user perceptions to reduce their potential health effects. Krüsemann et al [14] constructed an e-liquid flavor wheel based on flavor classifications using terms associated with e-cigarettes and flavors in research articles. Wang et al [15] investigated the e-cigarette content on Reddit without analyzing preferences and sentiments. A Twitter-related study focused on analyzing sentiments of general e-cigarette posts among different groups of users without considering e-cigarette flavors [16]. Currently, there have been few discussions about the e-liquid flavor classification, sentiment analysis, and trend analysis from the perspective of e-cigarette users on social media, which we explored in this study using Reddit and Twitter data.

With the emergence of new e-liquid flavors, the prevalence of mentions of e-liquid flavors might evolve over time, which needs to be monitored in a timely manner. In addition, it is important to understand how the perception of e-liquid flavors correlates with their prevalence. In this study, we employed a data-driven approach to characterize the dynamic changes in mentions of e-liquid flavors over time and to correlate them with the users’ perceptions of each flavor category on social media. With the development of technology, social media have become popular in our daily lives. The number of American adults using social networking sites increased from 7% in 2005 to almost 65% in 2015 and is still increasing [17]. We collected data from Reddit, which is one of the most popular social media platforms [18]. Reddit posts usually contain more information because they have a much higher character limit compared with other social media platforms such as Twitter, thus allowing the discussions on Reddit to focus more on the opinions and experiences of users. Twitter posts have a lower character limit than Reddit posts. This advantage makes it easier to locate flavor-related sentences, which can lead to a more accurate sentiment analysis compared with using Reddit data. Therefore, we downloaded Twitter data using the public Twitter app programming interface to explore people’s attitudes toward each flavor category. This study investigated the trends of mentions of e-liquid flavors and their perception on social media and provided some guidance for future research on the associations between the prevalence of e-cigarette flavors and user perceptions.

## Methods

### Electronic Cigarette Liquid Flavor Data Collection and Preprocessing

To obtain a full list of e-liquid flavor names, we searched the 3 major e-cigarette web-based stores, including *electrictobacconist.com, myvaporstore.com,* and *ecig-city.com*. The information on e-liquid flavors, including e-liquid brands, flavor names, and flavor ingredients, was manually recorded and curated. In addition to the unique flavor names (brand flavor names) from e-cigarette companies (eg, *Lava Flow* from *Naked 100* and *Green Goblin Salt* from *Oh My Gush*), specific flavor names (eg, mango, cotton candy, coffee) were also added to the list as people tend to mention specific flavor names more frequently on social media instead of using specific brand names. A total of 129 e-liquid brands and 1198 e-liquid flavors were collected.

### Electronic Cigarette Liquid Flavor Classification

We applied a data-driven approach to construct a hierarchical e-liquid flavor classification, including 3 levels: major flavor categories (column 1 in Table 1), subcategories (column 2 in Table 1), and specific flavors (column 3 in Table 1). Specific flavor names were first standardized based on the flavor components and key ingredients because different brands or websites may use different names for the same flavor. As some specific e-cigarette flavor names mentioned in Reddit posts were sparse, the specific flavors were classified into several major categories. Similar to categories mentioned in the study by Krüsemann et al [14], 7 major flavor categories were generated: fruit, tobacco, menthol or mint, sweets, beverage, mixed, and others (Multimedia Appendix 1). Within each major flavor category, specific flavors were merged into several subcategories (middle level). For example, in the *fruit* flavor category, there are *tropical*, *berry*, *melon*, *mixed fruit*, and *others* flavor subcategories. In each subcategory, specific fruit flavors were listed. E-liquid flavors that contain more than one flavor ingredient in multiple categories were classified into the *mixed* category. All other flavors that could not be classified into previous categories were classified into the *others* flavor category.

**Table 1:** Classification of electronic cigarette liquid flavors.

Flavor categories and subcategories		List of specific flavors
**Fruit**		
	Berry	Wildberry, currant, blackcurrant, blackberry, grape, raspberry, blueberry, strawberry, etc
	Tropical	Mango, lychee, guava, passion fruit, pineapple, etc
	Citrus	Grapefruit, lime, orange, lemon, etc
	Melon	Cantaloupe, honeydew, melon, watermelon, etc
	Mixed fruits	Mango apricot, apple melon, nana berry, etc
	Others	Pomelo, papaya, apricot, dragon fruit, pomegranate, cucumber, kiwi, pear, cherry, peach, coconut, banana, apple, etc
**Sweets**		
	Dessert	Mochi, pie, waffle, donut, mixed, cake, s’more, muffin, ice cream, cream, custard, macaron, granola, pastry, meringue, bread, cheesecake, cookie, etc
	Candy	Lollypop, mixed, jelly bean, gummy bear, cotton candy, marshmallow, bubble gum, chocolate, etc
	Others	Cereal, honey, caramel, etc
**Beverage**		
	Coffee	Latte, mocha, cappuccino, espresso, coffee
	Tea	Chai, tea, etc
	Juice	Limeade, lemonade, apple juice, etc
	Milk	Yogurt, milkshake, milk
	Soft drinks	Cola, coke, soda, etc
	Others	Energy drink, smoothie, etc
**Tobacco**		
	Tobacco	Classic tobacco, Virginia tobacco, cigar, etc
**Menthol or mint**		
	Menthol	Menthol
	Mint	Mint, peppermint, spearmint
**Mixed**		
	Mixed	Fruit + mint, fruit + sweets, fruit + beverage, fruit + beverage, sweets + other, sweets + mint, sweets + tobacco, fruit + tobacco, etc
**Others**		
	Alcohol	Margarita, whiskey, rum, bourbon, cocktail, etc.
	Nuts	Walnut, pecan, pistachio, hazelnut, almond, peanut butter, etc
	Spice	Vanilla, cinnamon, etc
	Others	Pure VG^a^, pure PG^b^, PG/VG, etc

^a^VG: vegetable glycerin.

^b^PG: propylene glycol.

### Reddit

#### Reddit Data Collection and Preprocessing

Reddit posts from January 2013 to April 2019 were downloaded from *pushshift.io* (a website that publishes archive Reddit data) [19]. E-cigarette–related Reddit posts were obtained using keyword matching based on a list of e-cigarette–related keywords, including *e-cig*, *e-cigs*, *ecig*, *ecigs*, *electroniccigarette*, *ecigarette*, *ecigarettes*, *vape*, *vapers*, *vaping*, *vapes*, *e-liquid*, *ejuice*, *eliquid*, *e-juice*, *vapercon*, *vapeon*, *vapefam*, *vapenation*, and *juul* [20]. As a result, 2,865,467 e-cigarette–related Reddit posts were collected.

We further extracted e-liquid flavor–related Reddit posts from the e-cigarette dataset we collected. We applied the constructed flavor classification scheme and used the flavors and flavor names as the keywords to obtain posts that discuss e-liquid flavors. We observed that some flavor names were very likely to cause confusion and introduce noises such as *contact* and *punched*; thus, a denoise procedure was performed to filter out noisy flavor keywords [21]. For each confusing flavor name, sample posts were generated and manually labeled. The goal of the denoise procedure was to optimize the precision to 90% while maintaining an acceptable recall of ≥75%. If flavor names did not achieve the goal, they were excluded from further data analysis. After filtering out noisy flavor keywords, the final flavor subset contained 904,045 posts (Multimedia Appendix 2).

#### Temporal Analysis of Reddit

A temporal analysis was conducted to investigate the longitudinal trend of the intensity of discussion about each e-cigarette flavor category using the monthly post counts from January 2013 to April 2019. To further investigate competitions and substitutions between flavors, data were further normalized by the total number of flavor subset post counts in each month.

### Twitter

#### Twitter Data Collection and Preprocessing

Using the same e-cigarette–related keywords, Twitter streaming data were downloaded from May 31 to August 22, 2019, leading to a collection of 2,757,860 e-cigarette–related Twitter posts.

To obtain e-cigarette flavor–related feedback from e-cigarette users instead of e-cigarette promotions from official accounts, promotion-related Twitter posts were filtered out in 2 steps. First, Twitter IDs that contain promotion-related keywords were eliminated, including *dealer*, *deal*, *store*, *supply*, *e-cig*, *e-cigs*, *ecig*, *ecigs*, *electroniccigarette*, *ecigarette*, *ecigarettes*, *vape*, *vapers*, *vaping*, *vapes*, *e-liquid*, *ejuice*, *eliquid*, *e-juice*, *vapercon*, *vapeon*, *vapefam*, *vapenation*, and *juul*. Second, Twitter posts that contain promotion-like keywords (eg, customer, promotion, discount, sale, and free shipping) were eliminated. After these 2 filtering procedures, a subset of 2,530,048 e-cigarette–related posts remained. To obtain an accurate result on the Twitter sentiment analysis of each flavor category, another flavor-related filtering was conducted by using keywords from the previous e-liquid flavor list Multimedia Appendix 1). As a result, we obtained an e-liquid flavor–related Twitter subset that contains 21,389 posts, and each post contains only 1 flavor keyword. The complete procedures of Twitter data preprocessing are shown in Multimedia Appendix 2.

#### Sentiment Analysis of Electronic Cigarette Liquid Flavors on Twitter

A sentiment analysis is a contextual analysis of sentences and paragraphs, which can extract subjective attitudes and opinions from the source material. The Valence Aware Dictionary and sEntiment Reasoner was used as the sentiment analyzer to extract the thoughts and opinions of e-cigarette users on each flavor category from Twitter posts [22]. We obtained a sentiment score for each tweet, and the average sentiment score was calculated for each flavor category. On the basis of the suggested threshold for determining the sentiment (positive, neutral, and negative), tweets with sentiment scores in the range of −1.00 to −0.05 were classified as negative posts, tweets with sentiment scores in the range of −0.05 to +0.05 (not include −0.05 and +0.05) were classified as neutral posts, and tweets with sentiment scores in the range of +0.05 to +1.00 were classified as positive posts [22]. The number of posts for each flavor category with positive, neutral, and negative sentiments was normalized by the total number of posts for each flavor category to explore the distribution of the sentiment results for each flavor category. In addition, comparisons between the proportions of positive and negative Twitter posts within each flavor category were conducted using 2 proportion z tests in SAS version 9.4 (SAS Institute Inc) to determine the significant differences between the proportion of positive sentiments and the proportion of negative sentiments. All tests were two sided, with a significance level of 5%. The original *P* values were adjusted using the Bonferroni method to account for multiplicity.

## Results

### Prevalence of Electronic Cigarette Flavors Mentioned on Reddit

E-liquid flavors were classified into different flavor categories. Table 1 provides an overview of the multiple levels of classifications, including major flavor categories, subcategories, and specific flavors. The proportions of hierarchical 3-layer flavor classifications are shown in Multimedia Appendix 1. Using the filtered Reddit data, Table 2 shows the percentage distribution of major flavor categories as well as detailed percentage distributions of 4 major flavor subcategories. As shown in Table 2, the *fruit* and *sweets* categories were the most mentioned and popular flavor categories on Reddit, followed by *beverage*, *menthol or mint*, *tobacco*, *others,* and *mixed*. In addition, *berry* was the dominant subcategory with a percentage of 45.84% (7,27,404/15,86,926) over all fruit-related subcategories; *dessert* was dominant in the *sweets* subcategory with a percentage of 47.43% (1,89,946/400,500). *Coffee* (118,129/2,77,005, 42.65%) and *tea* (1,07,449/2,77,005, 38.79%) were the most popular subcategories in the *beverage* flavor category. In the *menthol or mint* flavor category, the *menthol* (1,73,641/2,29,817, 75.56%) subcategory was more popular than the *mint* (56,176/2,29,817, 24.44%) subcategory. The percentage of post count for specific flavors in Multimedia Appendix 1 showed that the *strawberry* flavor was the most mentioned in the *fruit* category, and the *vanilla* flavor had the most mentions in the s*weets* category.

**Table 2:** Percentage distribution of e-liquid flavors mentioned on Reddit.

Flavor categories and subcategories		Count, n (%)
**Fruit**		1,586,926 (58.15)
	Berry	727,404 (45.84)
	Others	374,886 (23.62)
	Tropical	190,639 (12.01)
	Melon	138,876 (8.75)
	Mixed fruit	102,618 (6.47)
	Citrus	52,503 (3.31)
**Sweets**		400,500 (14.67)
	Dessert	189,946 (47.43)
	Others	113,688 (28.39)
	Candy	96,866 (24.18)
**Beverage**		277,005 (10.15)
	Coffee	118,129 (42.64)
	Tea	107,449 (38.80)
	Milk	21,764 (7.85)
	Juice	21,117 (7.62)
	Soft drink	5377 (1.94)
	Others	3169 (1.14)
**Menthol or mint**		229,817 (8.42)
	Menthol	173,641 (75.56)
	Mint	56,176 (24.44)
Tobacco		163,377 (5.99)
Others		43,922 (1.61)
Mixed		24,769 (0.91)

### Temporal Analysis of Mentions of Electronic Cigarette Liquid Flavors on Reddit

**Figure 1:**
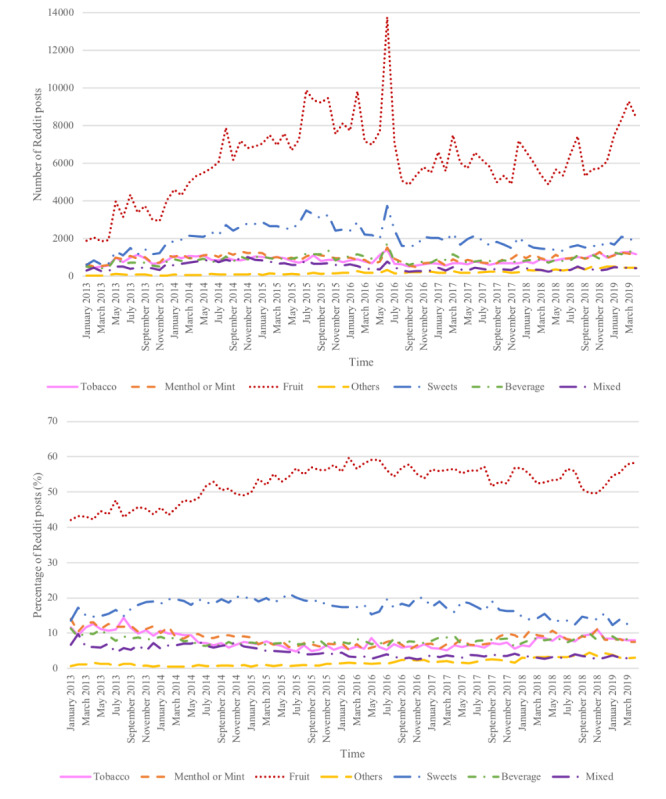
The longitudinal trend of e-liquid flavors mentioned on Reddit, including the number of Reddit posts and the proportion of Reddit posts of each flavor category from January 2013 to April 2019. The proportion is normalized by the monthly total flavor-related post count. e-liquid: electronic cigarette liquid.

With the availability of new e-liquid flavors and the changes in the perception of users on e-liquid flavors over time, it is important to examine the temporal changes of e-liquid flavors mentioned on social media, so that the appropriate regulation of e-liquid flavors could be applied. To address this, we used the Reddit data collected from January 2013 to April 2019. By examining the number of Reddit posts on each e-liquid flavor, we observed a relatively increasing trend on the *fruit* category, whereas it remained relatively constant for the other flavor categories (Figure 1). The temporal analysis also showed a significant spike for each flavor category in June 2016 (Figure 1). This spike coincided with the US Food and Drug Administration (FDA), extending its regulatory authority to all tobacco products, including e-cigarettes and pipe tobacco on June 16, 2016. The number of posts mentioning e-liquid flavors on Reddit after June 2016 was much less than that before June 2016 but still showed an upward trend. After normalization to all posts mentioning flavors, the *fruit* flavor category had a steady upward trend from January 2013 to June 2016 and then showed some fluctuation. Most of the other flavor categories did not show significant trends, with *sweets* and *mixed* categories having a relatively noticeable decreasing trend.

### Sentiment Analysis of Electronic Cigarette Liquid Flavors Mentioned on Twitter

Although there are more than 7000 e-liquid flavors available on the market, it is important to examine the perception of public users to different e-liquid flavors, which will help us understand their prevalence on social media. As the character limit of Reddit subreddits is 40,000, it was difficult to locate the sentences only about e-liquid flavors in long Reddit messages. In this case, sentiment analysis precision for e-liquid flavors on Reddit cannot be ensured. Thus, Twitter data with a character limit of 280 were used instead for the sentiment analysis for more precise results. To obtain the most recent sentiments toward e-liquid flavors, we decided to use Twitter data from May 31 to August 22, 2019. As the total post counts in the *others* (276 posts) and the *mixed* (126 posts) flavor categories are much lower than other flavor categories and because e-cigarette flavors in these 2 categories were miscellaneous and diverse, we excluded these 2 categories in the sentiment analysis. The sentiment analysis on each e-liquid flavor category using Twitter data showed that, on average, the posts with the *sweets*, *menthol or mint*, and *fruit* flavor categories had positive sentiments, whereas the posts with the *tobacco* and *beverage* flavor categories showed negative sentiments (Table 3).

**Figure 2:**
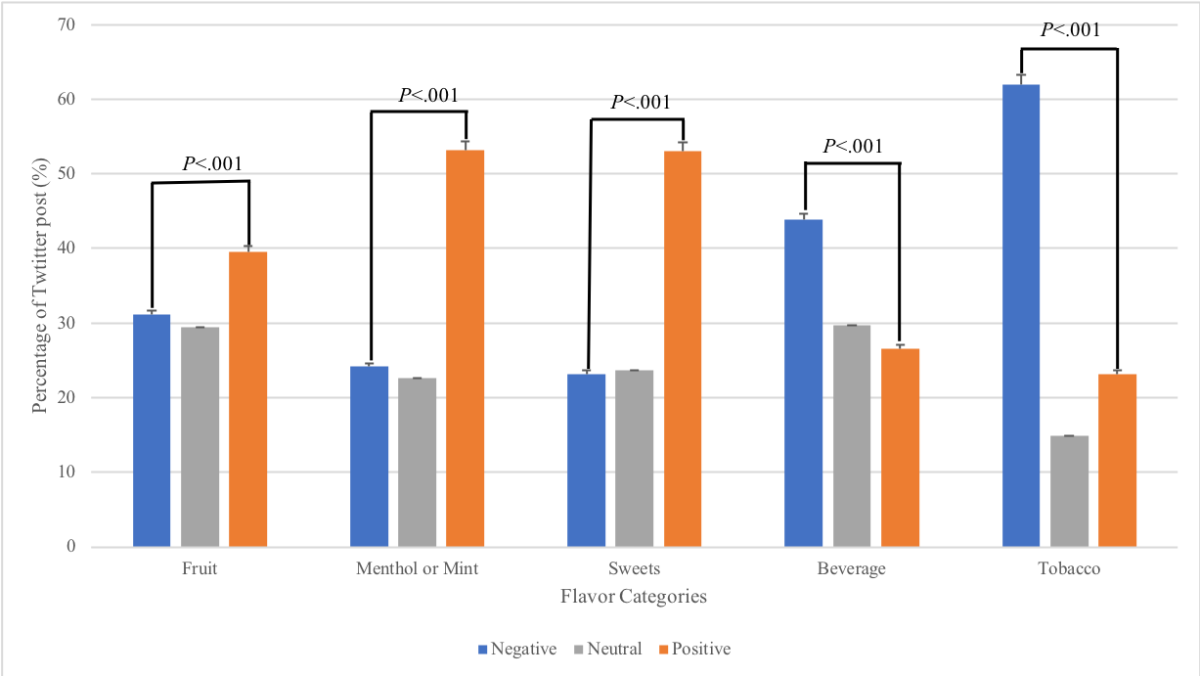
Sentiment analysis of the flavor categories mentioned on Twitter. Error bars represent the estimated SDs.

On the basis of thresholds of +/- 0.05 for positive and negative sentiments, each post was classified into positive, negative, or neutral sentiments. Within each flavor category, the proportion of posts with different sentiments was calculated (Figure 2). The *fruit*, *menthol or mint*, and *sweets* flavor categories had a significantly higher proportion of positive posts than negative posts (*P*<.001). In contrast, the *beverage* and *tobacco* flavor categories had a significantly higher proportion of negative posts than positive posts (*P*<.001).

**Table 3:** Sentiment analysis of flavor categories on Twitter.

Flavor category	Total post count, N	Average sentiment score, mean (SD)
Fruit	9852	0.074 (0.453)
Menthol or mint	4582	0.128 (0.379)
Sweets	3190	0.156 (0.442)
Beverage	1805	−0.090 (0.451)
Tobacco	1555	−0.134 (0.440)

## Discussion

### Principal Findings

In this study, by mining e-cigarette–related posts on social media (Reddit and Twitter), we showed that *fruit* and *sweets* flavors were the most popular flavors mentioned on social media, which remained to be the top popular flavors mentioned on social media over time. In addition, the sentiment analysis result showed significant positive attitudes toward *fruit* and s*weets* flavor categories. Although there were significant negative attitudes toward *tobacco* and *beverage* flavor categories, these 2 flavors were less mentioned on social media. Therefore, the perception of e-cigarette flavors on social media positively correlates with their popularity, that is, a positive sentiment correlates with high popularity on social media.

Our temporal studies showed that *fruit* and *sweets* have constantly been the most frequently mentioned e-liquid flavors on social media from 2013 to 2019, suggesting that the prevalence of e-liquid flavors mentioned on social media has not changed much over time. However, with the implementation of the e-cigarette flavor enforcement policy by the FDA on February 6, 2020, which bans all cartridge-based–flavored e-cigarettes except tobacco and menthol flavors, the popularity of e-liquid flavors will change accordingly. In addition, with the availability of other flavored e-cigarette products (such as disposable e-cigarette devices) on the market after the FDA flavor enforcement policy, it is hard to predict which flavors will become popular, which warrants further investigation.

In this study, we provided a way to timely monitor the prevalence of e-liquid flavors mentioned on social media as well as to identify the positive correlation between the perception of e-liquid flavors and their prevalence. With the power of this surveillance system, current regulation on flavored e-cigarettes could be modified or updated in a timely manner, and more importantly, the epidemic of e-cigarette use, especially among the youth, could be ameliorated to protect public health.

### Comparison With Prior Work

Compared with the previous flavor wheel that was based on manually reviewed papers, the proposed e-liquid flavor classification in this study has better coverage of the up-to-date e-cigarette flavors with practical frequency distributions because it was constructed based on products on the web and discussions on social media [14]. As a result, we revised the flavor classification in several ways. First, the major flavor categories were merged from 13 into 7 categories [14]. For example, we combined the *candy*, *dessert*, and *other sweets* categories into the other sweets category of *sweets* as most of these flavors have sweetener ingredients. In addition, subcategories were created to form a more detailed flavor classification. For example, within the *fruit* flavor category, besides the existing second-level subcategories *tropical*, *berry*, *citrus*, and *others*, a *melon* subcategory was added because of the frequent mentions of melon-related flavors on social media. Together, the revised flavor classification contains more complete and the most up-to-date e-cigarette flavors.

A previous study showed the prevalence of the mentions of e-liquid flavor use on Reddit [15]. Instead of showing the yearly nominal post counts of each flavor category, our temporal analysis used the total flavor-related Reddit post counts to normalize the monthly data to see the trend of mentions of e-liquid flavors. Similar to previous findings [15], our results showed that the *fruit* flavor category was the dominant flavor on Reddit, followed by *sweets*. Another study showed that the *fruit* flavor category was mentioned most frequently on Facebook, followed by *sweet* and *cream* (classified as the *sweets* flavor category in our study) [23]. Compared with the findings from the previous study [15], the *beverage* flavor had a slightly larger portion of mentions recently. The percentage distribution of the *beverage* flavor increased from 7% to 10% from 2015 to 2019. Although this increase is small, which could be because of random error, there might be 2 other reasons. First, it could be caused by increased discussions about the *beverage* flavors on Reddit. Second, our *beverage* flavor-filtering keyword list contains more flavors. Besides the *coffee* and *tea* flavors, we added the *juice*, *soft drinks*, and *energy drinks* flavor subcategories in the *beverage* category, which might cause the *beverage* flavor category to have a larger percentage of mentions on Reddit [15].

On June 16, 2016, the FDA finalized a rule that extended its regulatory authority to all tobacco products, including e-cigarettes and pipe tobacco, as part of its goal to improve public health. This new rule also restricted youth access to newly regulated tobacco products [24]. Due to this new rule released by the FDA, more flavor-related e-cigarette Reddit posts were mentioned during that month, causing a significant spike in Figure 1. These data also demonstrated that social media data could accurately and timely reflect what happens in real life. There was a slightly increasing trend in the *fruit* category in Figure 1, which suggests that people tend to talk about fruit-related e-liquid flavors more frequently on Reddit compared with other flavor categories. A recent study using the Population Assessment of Tobacco and Health Wave 3 data showed that 52.8% of youths used fruit-flavored electronic nicotine delivery systems, which is consistent with our findings on the prevalence of fruit flavors (58%) mentioned using Reddit data [25]. Our sentiment analysis showed positive attitudes toward the fruit flavor category. Thus, our data suggest that the regulation of e-cigarette use should focus on the restriction of e-cigarette use with fruit flavors.

There have been few studies on flavor sentiment analysis on social media [16,26]. One study focused on e-cigarette–related Twitter post sentiments among users with respect to different genders and age ranges [16]. However, flavor-related sentiments were not discussed. Another study showed the sentiments on e-liquid flavors mentioned on JuiceDB, but only focusing on the 2 most popular flavors, *fruit* and *sweet* flavors [26]. Our results were similar to the sentiment analysis from JuiceDB, showing that the overall sentiments for those 2 flavors were positive. More importantly, our results performed a sentiment analysis on other flavor categories, thus providing a more comprehensive picture of the perception of e-liquid flavors on social media.

### Limitations

In this study, Reddit and Twitter data were used to answer different questions considering their characteristics. A Reddit post has a relatively large character limit, which makes it an ideal data source to examine the experience users have with e-cigarettes. In addition, Reddit posts were available from January 2013 to April 2019, which made it possible to examine the temporal changes in popular e-liquid flavors. However, as Reddit posts have a 40,000-character limit, it is difficult to obtain accurate sentiment results for specific e-liquid flavors. Twitter posts have a much smaller character limit (280 characters) compared with Reddit, which makes it ideal for a sentiment analysis. Switching social media platforms from Reddit to Twitter in the process of a sentiment analysis may create some bias. The attitudes of users toward e-cigarette flavors on Twitter might be slightly different from those on Reddit.

Although there are more than 7000 e-liquid flavors on the market [27], just over 1000 specific e-liquid flavors were included in our dataset. In the future, more e-liquid brands and flavors will be collected for a more complete dataset, which can generate more detailed flavor classification. More analyses could be conducted by using additional social media platforms, such as Facebook and Instagram.

User demographic information, including gender, age, and ethnicity, is not available from social media data, which might limit further analysis of the prevalence of e-liquid flavors mentions among different demographic groups.

In general, the information from social media data is noisy, which could bias the results. However, our results are consistent with the results from the national survey data, which indicate the reliability of our conclusions.

With the announcement of the e-cigarette flavor enforcement policy by the FDA at the beginning of 2020, the prevalence of e-cigarette flavors will likely evolve as most flavors (such as fruit and sweets) are banned and as disposable e-cigarette devices with different flavors become available on the market. Understanding which flavors are the most popular after the flavor enforcement policy will be very important, which awaits further investigation.

### Conclusions

Although the popularity of e-liquid flavors has been reported in previous studies, this study showed the longitudinal changes in the prevalence of mentions of e-liquid flavors as well as the perceptions users have of different e-liquid flavors. The findings from this study will provide the most updated information about the popular e-liquid flavors mentioned on social media and how the perceptions of different e-liquid flavors are correlated with their prevalence on social media, which could guide further regulation of e-cigarette flavors. Our results showed that the prevalence and perceptions of different flavors on social media were different. The findings of this study have several valuable applications. Social media data can be used to inform researchers or policymakers about the prevalence of mentions of different e-liquid flavors in a timely manner and provide some guidance on the regulation of flavored e-cigarettes. Although the FDA has restricted the sale of unauthorized flavored cartridge-based e-cigarettes, flavored e-cigarettes in other formats still exist in the market, such as disposable e-cigarette devices. Here, we provided a cost-effective surveillance system to monitor the prevalence of e-liquid flavors over time.

## Appendix

Multimedia Appendix 1 Flowchart of Data Pre-processing Procedures.

Multimedia Appendix 2 E-liquid Flavors mentioned on Reddit.

## Abbreviations

e-cigarette: electronic cigarette
e-liquid: electronic cigarette liquid
FDA: Food and Drug Administration
