# Supplementary figures and images for: User Perceptions of Different Electronic Cigarette Flavors on Social Media: Observational Study
Source: J Med Internet Res. 2020 Jun 24;22(6):e17280. doi: 10.2196/17280 (PMC7380993; doi:10.2196/17280)

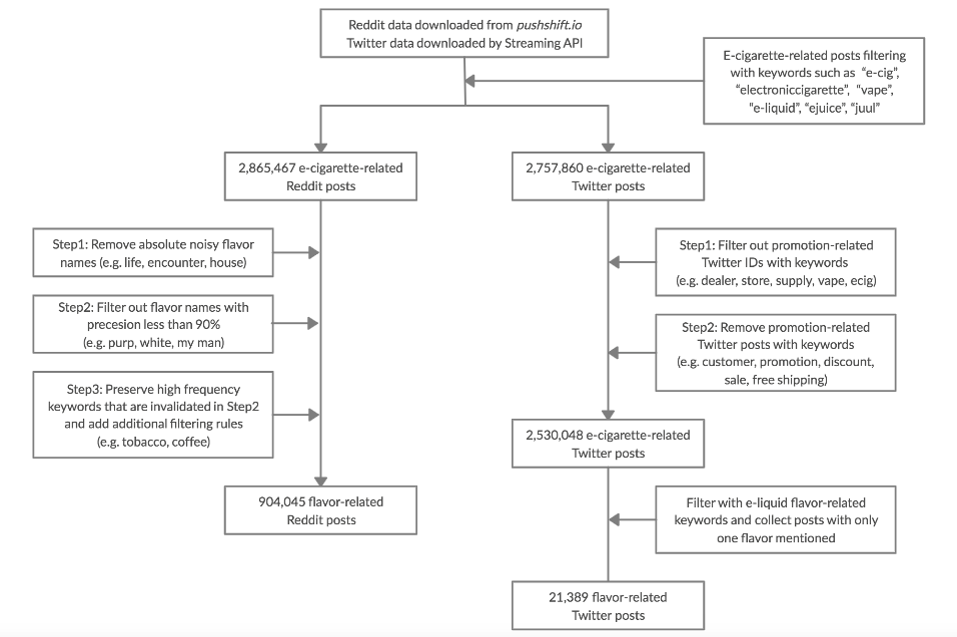

Supplement: Multimedia Appendix 1 [file jmir_v22i6e17280_app1.png]
