# Supplementary material for: User Perceptions of Different Electronic Cigarette Flavors on Social Media: Observational Study
Source: J Med Internet Res. 2020 Jun 24;22(6):e17280. doi: 10.2196/17280 (PMC7380993; doi:10.2196/17280)
Supplement: Multimedia Appendix 2 [file jmir_v22i6e17280_app2.docx]

**Supplemental Table 1. E-liquid Flavors mentioned on Reddit**

| E-liquid Flavor Categories | E-liquid Subcategories | E-liquid Flavors | Total Post Count | Percentage of Post Count |
| --- | --- | --- | --- | --- |
| Fruit | Berry | Strawberry | 431,527 | 15.811% |
|  |  | Others | 114,090 | 4.181% |
|  |  | Grape | 84,736 | 3.105% |
|  |  | Blueberry | 78,464 | 2.875% |
|  |  | Raspberry | 13,387 | 0.490% |
|  |  | Blackberry | 5,200 | 0.191% |
|  | Others | Apple | 195,271 | 7.155% |
|  |  | Cherry | 53,274 | 1.952% |
|  |  | Peach | 52,440 | 1.921% |
|  |  | Coconut | 23,992 | 0.879% |
|  |  | Banana | 21,226 | 0.778% |
|  |  | Pear | 12,847 | 0.471% |
|  |  | Cucumber | 6,072 | 0.222% |
|  |  | Kiwi | 4,212 | 0.154% |
|  |  | Others | 2,206 | 0.081% |
|  |  | Pomegranate | 883 | 0.032% |
|  |  | Dragon Fruit | 662 | 0.024% |
|  |  | Apricot | 598 | 0.022% |
|  |  | Blackcurrant | 446 | 0.016% |
|  |  | Currant | 424 | 0.016% |
|  |  | Papaya | 333 | 0.012% |
|  | Tropical | Mango | 157,411 | 5.767% |
|  |  | Pineapple | 16,782 | 0.615% |
|  |  | Others | 8,274 | 0.303% |
|  |  | Passion Fruit | 4,603 | 0.169% |
|  |  | Lychee | 2,453 | 0.090% |
|  |  | Guava | 1,116 | 0.041% |
|  | Melon | Watermelon | 90,712 | 3.324% |
|  |  | Melon | 42,928 | 1.573% |
|  |  | Honeydew | 4,749 | 0.174% |
|  |  | Cantaloupe | 483 | 0.018% |
|  |  | Others | 4 | 0.000% |
|  | Citrus | Lemon | 25,242 | 0.925% |
|  |  | Orange | 15,536 | 0.569% |
|  |  | Lime | 5,814 | 0.213% |
|  |  | Others | 4,409 | 0.162% |
|  |  | Grapefruit | 1,502 | 0.055% |
|  | Mixed Fruit | Mixed Fruit | 102,618 | 3.760% |
| Sweets | Dessert | Cream | 74,499 | 2.730% |
|  |  | Pie | 35,041 | 1.284% |
|  |  | Custard | 30,381 | 1.113% |
|  |  | Cake | 12,265 | 0.449% |
|  |  | Cereal | 6,611 | 0.242% |
|  |  | Others | 5,990 | 0.219% |
|  |  | Cookie | 5,525 | 0.202% |
|  |  | Ice Cream | 4,801 | 0.176% |
|  |  | Muffin | 3,158 | 0.116% |
|  |  | Donut | 3,016 | 0.111% |
|  |  | Cheesecake | 2,983 | 0.109% |
|  |  | Bread | 2,696 | 0.099% |
|  |  | Meringue | 1,053 | 0.039% |
|  |  | Pastry | 771 | 0.028% |
|  |  | S'more | 635 | 0.023% |
|  |  | Mixed | 295 | 0.011% |
|  |  | Granola | 226 | 0.008% |
|  | Others | Vanilla | 113,021 | 4.141% |
|  |  | Others | 667 | 0.024% |
|  | Candy | Bubble Gum | 31,499 | 1.154% |
|  |  | Candy | 20,737 | 0.760% |
|  |  | Chocolate | 19,059 | 0.698% |
|  |  | Caramel | 11,936 | 0.437% |
|  |  | Cotton Candy | 8,840 | 0.324% |
|  |  | Marshmallow | 1,945 | 0.071% |
|  |  | Gummy Bear | 2,053 | 0.075% |
|  |  | Others | 797 | 0.029% |
| Beverage | Coffee | Coffee | 112,026 | 4.105% |
|  |  | Latte | 2,906 | 0.106% |
|  |  | Cappuccino | 1,947 | 0.071% |
|  |  | Espresso | 636 | 0.023% |
|  |  | Mocha | 614 | 0.022% |
|  | Tea | Tea | 64,028 | 2.346% |
|  |  | Chai | 43,421 | 1.591% |
|  | Milk | Milk | 20,148 | 0.738% |
|  |  | Yogurt | 1,616 | 0.059% |
|  | Juice | Lemonade | 19,516 | 0.715% |
|  |  | Others | 1,015 | 0.037% |
|  |  | Apple Juice | 388 | 0.014% |
|  |  | Limeade | 198 | 0.007% |
|  | Soft Drinks | Others | 3,460 | 0.127% |
|  |  | Cola | 1,917 | 0.070% |
|  | Others | Milkshake | 1,305 | 0.048% |
|  |  | Smoothie | 986 | 0.036% |
|  |  | Others | 878 | 0.032% |
| Menthol/Mint | Menthol | Menthol | 173,641 | 6.362% |
|  | Mint | Mint | 54,091 | 1.982% |
|  |  | Peppermint | 2,085 | 0.076% |
| Tobacco | Tobacco | Tobacco | 163,115 | 5.976% |
|  |  | Cigar | 262 | 0.010% |
| Others | Alcohol | Rum | 16,208 | 0.594% |
|  |  | Bourbon | 1,338 | 0.049% |
|  |  | Whiskey | 1,100 | 0.040% |
|  |  | Others | 189 | 0.007% |
|  | Spice | Cinnamon | 9,084 | 0.333% |
|  |  | Others | 6,217 | 0.228% |
|  | Nuts | Peanut Butter | 2,628 | 0.096% |
|  |  | Almond | 1,320 | 0.048% |
|  |  | Hazelnut | 1,120 | 0.041% |
|  |  | Pistachio | 546 | 0.020% |
|  |  | Pecan | 427 | 0.016% |
|  |  | Walnut | 346 | 0.013% |
|  | Others | Others | 3,399 | 0.125% |
| Mixed | Mixed | Mixed | 24,769 | 0.908% |
